# Intermittent Compressive Stress Enhanced Insulin-Like Growth Factor-1 Expression in Human Periodontal Ligament Cells

**DOI:** 10.1155/2015/369874

**Published:** 2015-05-28

**Authors:** Jittima Pumklin, Jeeranan Manokawinchoke, Kanokporn Bhalang, Prasit Pavasant

**Affiliations:** ^1^Research Unit of Mineralized Tissue, Faculty of Dentistry, Chulalongkorn University, Bangkok 10330, Thailand; ^2^Graduate Program in Oral Biology, Faculty of Dentistry, Chulalongkorn University, Bangkok 10330, Thailand; ^3^Department of Anatomy, Faculty of Dentistry, Chulalongkorn University, Bangkok 10330, Thailand; ^4^Department of Oral Medicine, Faculty of Dentistry, Chulalongkorn University, Bangkok 10330, Thailand

## Abstract

Mechanical force was shown to promote IGF-1 expression in periodontal ligament both *in vitro* and *in vivo*. Though the mechanism of this effect has not yet been proved, here we investigated the molecular mechanism of intermittent mechanical stress on *IGF-1* expression. In addition, the role of hypoxia on the intermittent compressive stress on *IGF-1* expression was also examined. In this study, human periodontal ligament cells (HPDLs) were stimulated with intermittent mechanical stress for 24 hours. *IGF-1* expression was examined by real-time polymerase chain reaction. Chemical inhibitors were used to determine molecular mechanisms of these effects. For hypoxic mimic condition, the CoCl_2_ supplementation was employed. The results showed that intermittent mechanical stress dramatically increased *IGF-1* expression at 24 h. The pretreatment with TGF-*β* receptor I or TGF-*β*1 antibody could inhibit the intermittent mechanical stress-induced *IGF-1* expression. Moreover, the upregulation of TGF-*β*1 proteins was detected in intermittent mechanical stress treated group. Correspondingly, the *IGF-1* expression was upregulated upon being treated with recombinant human TGF-*β*1. Further, the hypoxic mimic condition attenuated the intermittent mechanical stress and rhTGF-*β*1-induced *IGF-1* expression. In summary, this study suggests intermittent mechanical stress-induced *IGF-1* expression in HPDLs through TGF-*β*1 and this phenomenon could be inhibited in hypoxic mimic condition.

## 1. Introduction

In oral cavity, mechanical stress was generated in many situations, such as mastication, functional/parafunctional habits, orthodontic treatment, and occlusal trauma. Occlusal force plays a pivotal role in the regulation of periodontium homeostasis [1–3]. The mechanical force in the range of physiological condition was involved in the maintaining of the periodontium system [4]. However, the force exceeding physiological limitation could lead to pathological change, such as periodontal ligament (PDL) space widening, periodontium destruction, and alveolar bone resorption [5, 6].

Several lines of evidence demonstrated the effect of mechanical stress on cellular response, including periodontal ligament cells (PDLs) [4, 7]. It has been illustrated both* in vitro* and* in vivo* that mechanical stress influenced PDL behavior. Several techniques were employed to investigate the effect of mechanical stress* in vitro*, for example, shear stress [8], cyclic tensile stress [9, 10], and static compressive stress [11]. The previous data showed that PDL responded to mechanical stress by releasing ATP [12, 13], increasing intracellular calcium [11, 14], changing actin filament organization [15], and upregulating of several cytokines or growth factor, including insulin-like growth factor-1 (IGF-1) [16–18].

IGFs consist of several family members such as IGF-1 and IGF-2 [19]. IGF-1 plays a role in various cellular activities, including survival, proliferation, and differentiation [20–26]. IGF-1 is involved in several kinds of cells and tissues [19] while IGF-2 plays an important role mainly during prenatal development [21]. It has been illustrated that human PDL expressed the IGF1 receptor, implying the ability to IGF-1 stimulation [27]. Previous report showed that IGF-1 enhanced human periodontal ligament cells (HPDLs) survival by inducing antiapoptotic molecules and downregulating proapoptotic molecules [22]. Furthermore, IGF-1 was shown to promote proliferation and osteogenic differentiation in human PDL [24]. It was noted that the application of orthodontic force on rat teeth resulted in the upregulated IGF-1 release in PDL* in vivo* [16, 18, 28], though the molecular mechanism, by which mechanical stress stimulates IGF-1 expression, is yet unclear.

Therefore, the present study aimed to investigate molecular signaling mechanism of intermittent mechanical stress on the* IGF-1* expression in human PDLs. Furthermore, the influence of hypoxia on the intermittent mechanical stress regulated* IGF-1* expression was examined.

## 2. Materials and Methods

### 2.1. Materials

Cell culture medium was purchased from Gibco BRL (BRL, Carlsbad, CA, USA). Culture dishes and plastic tubes were purchased from Corning (Corning, NY, USA). Cobalt chloride (CoCl_2_) was purchased from Santa Cruz Biotechnology Inc. (Santa Cruz, CA, USA). Cyclohexylamine, genistein, monensin, TGF-*β* receptor I inhibitor (SB431542), and recombinant human TGF-*β*1 (rhTGF-*β*1) were purchased from Sigma-Aldrich (St. Louis, MO, USA). P38 MAPK inhibitor (SB203580) was purchased from Calbiochem (Merck Chemicals, Gibbstown, NJ, USA). The TGF-*β*1 antibody was purchased from R&D Systems Inc. (Minneapolis, MN, USA).

### 2.2. Cell Culture

All protocol was approved by the Ethics Committee of the Faculty of Dentistry, Chulalongkorn University. Third molars and premolars extracted for orthodontic reasons at the Faculty of Dentistry, Chulalongkorn University, were collected for cell isolation. The periodontal tissue was obtained from middle third of teeth's root and the tissue was cultured in standard medium (Dulbecco's modified Eagle's medium (DMEM) containing 10% fetal bovine serum with 1% L-glutamine and 1% Ab/Am) and incubated at 37°C in a humidified atmosphere of 5% CO_2_ in air. After the cells migrated from the tissue and became confluent, they were detached with 0.25% trypsin-EDTA and subcultured at a 1 : 3 ratio. In each experiment, cells from at least 3 donors were used.

### 2.3. Hypoxic Mimic Condition

Hypoxic mimic condition was generated by the supplementation of CoCl_2_. Cells were incubated with CoCl_2_ at 150 or 300 *μ*M for 30 min prior to applying intermittent mechanical stress. The control groups were cultured in the absence of CoCl_2_.

### 2.4. Intermittent Mechanical Stress Treatment

A cell compressive force loading apparatus (Thai Patent ID: 1401006767) was designed and constructed to mechanically stimulate cells in a culture plate [29]. Cells were seeded in 6-well culture plates at a density of 3 × 10^5^ cells per well overnight. The cells were starved with serum-free culture medium for 4 h before loading force. Compressive force generator V2.5 software was used to set times, loading type, and the amount of force. In brief, the loading cycle was set to press for 1 s and to unpress for 2 s to yield a loading cycle approximately 1/3 Hertz and the force amount 1.5 g/cm^2^.

In some experiments, SB203580 (3.5 *µ*M), CoCl_2_ (150 *μ*M), cyclohexylamine (10 *μ*M), genistein (92.5 *μ*M), monensin (100 *μ*M), SB431542 (10 *μ*M), rhTGF-*β*1 (2 ng/mL), or TGF-*β*1 antibody (5 *μ*g/mL) was added in the culture condition.

### 2.5. Cell Viability

HPDLs were seeded in 6-well plates at a density of 3 × 10^5^ cells per well for applying the force and 24-well plates at density of 5 × 10^4^ cells per well for being treated with CoCl_2_. Subsequently cells were starved with serum-free media 4 h before treatment. At 24 h, HPDLs were incubated with 3-(4, 5-dimethylthiazol-2-yl)-2, 5-diphenyltetrazolium bromide solution for 30 min. Formazan crystals were solubilized in DMSO/glycine buffer solution (0.1 M glycine/0.1 M sodium chloride pH10). The solution was further measured for an absorbance at 570 nm in a microplate reader (Elx800, Biotek, USA). The data were normalized to the control. All measurements were done in triplicate.

### 2.6. Real-Time Polymerase Chain Reaction (Real-Time PCR)

After 24 h, total cellular RNA was extracted with Trizol reagent (Molecular research Center, Cincinnati, Ohio, USA) according to the manufacturer's instructions. RNA was quantified using a NanoDrop 2000 Spectrophotometer (Thermo Scientific, Wilmington, DE, USA). One microgram of RNA sample was converted to cDNA by ImProm-II (Promega, Madison, WI, USA). Subsequently, the real-time PCR reaction was using a LightCycler instrument (Roche Diagnostics, USA) with the LightCycler 480SYBR Green I Master kit according to the manufacturer's specifications. Relative gene expression was calculated by RelQuant software (Roche Diagnostics, USA). Gene expression was normalized to the 18S ribosome expression. The results are shown as fold-change values relative to the control group. The oligonucleotide sequences were as follows:* IGF-1* (NM000618.3), forward 5′-CATGCCTGCTCAGAAGGGTA-3′, reverse 5′-GCCTCTGATCCTTGAGGTGA-3′;* 18S* (NR003286.2), forward 5′-GGCGTCCCCCAACTTCTTA-3′, reverse 5′-GGGCATCACAGACCTGTTATT-3′.

### 2.7. Enzyme-Linked Immunosorbent Assay (ELISA)

Radioimmunoprecipitation assay (RIPA) supplemented with protease inhibitors was used to extract cellular protein. The amount of protein was measured by a BCA protein assay kit (Pierce, Rockford, IL). Whole cell lysate and condition medium were collected at −80°C for measuring the level of protein. ELISA was used for measuring the protein level according to the manuals of ELISA kits (Quantikine Immunoassay R&D Systems). The absorbance of ELISA reaction product was measured at OD 450 nm using microplate reader (BioTek, ELx800, USA).

### 2.8. Statistical Analyses

Data were reported as mean ± SD. Statistical analyses were performed for two independent samples using the Student* t*-test for two-group comparisons. A one-way analysis of variance (ANOVA) followed by Turkey's* post hoc* analysis (SPSS, Chicago, IL, USA) was employed for three or more group comparisons. The *p* value less than 0.05 was considered as statistically significant.

## 3. Results

### 3.1. Intermittent Mechanical Stress-Induced* IGF-1* Expression

**Figure 1:**
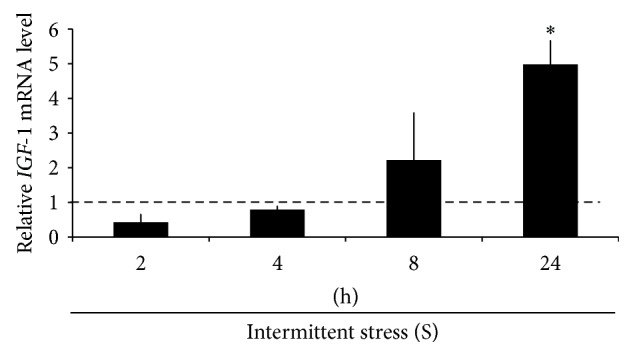
Intermittent mechanical stress-induced* IGF-1* expression. HPDLs were treated with intermittent mechanical stress for 2 h, 4 h, 8 h, and 24 h. The* IGF-1* mRNA expression was determined using real-time PCR. The dot line represented the expression levels of the control. Asterisks indicated statistically significant difference.

We began by investigating the effect of intermittent mechanical stress on HPDLs viability and morphology using a microscope at 100x magnification. HPDLs morphology was similar in all groups (see Supplementary Figure 1c in Supplementary Material available online at http://dx.doi.org/10.1155/2015/369874) and mechanical stress did not affect the HPDLs viability (Supplementary Figures 1a and 1b). Next, we investigated the effect of intermittent mechanical stress on* IGF-1* expression in HPDLs at different time points (Figure 1). There was no significant difference in* IGF-1* expression at 2 h, 4 h, or 8 h between the intermittent mechanical stress-treated group and the control group. However, the* IGF-1* mRNA levels were significantly increased at 24 h after exposing to mechanical stress. Thus, these results demonstrated intermittent mechanical stress-induced* IGF-1* expression in HPDLs at 24 h.

### 3.2. Intermittent Mechanical Stress Required Intermediate Protein to Induce* IGF-1* Expression

**Figure 2:**
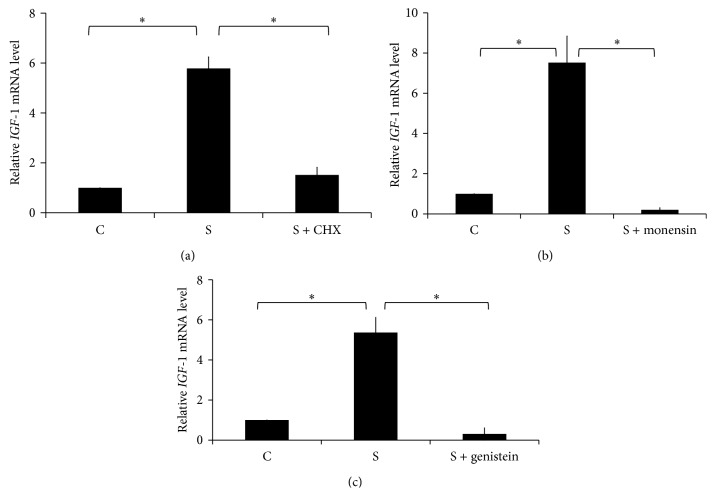
Intermittent mechanical stress required the intermediate protein to induce* IGF-1* expression. (a) Cycloheximide (CHX; 10 *μ*M), (b) genistein (92.5 *μ*M), and (c) monensin (100 *μ*M) were pretreated 30 min prior to applying the intermittent mechanical stress for 24 h.* IGF-1* mRNA expression was determined by real-time PCR. Asterisks indicated statistically significant difference. C: the control condition; S: the intermittent mechanical stress treatment condition.

We started to pretreat HPDLs with SB203580 which is p38 MAPK inhibitor prior to applying the force. Our results demonstrated that p38 MAPK inhibitor failed to block intermittent mechanical stress-induced* IGF-1* expression in HPDLs (Supplementary Figure 2). Also, cycloheximide was used to inhibit protein translation (Figure 2(a)). The results showed that cycloheximide pretreatment inhibited the intermittent compressive force-induced* IGF-1* mRNA expression. Further, the mechanical force-induced* IGF-1* expression was also inhibited by the monensin, a protein transport inhibitor (Figure 2(b)). These results imply that the intermittent mechanical stress required the release of intermediate protein to induce* IGF-1* expression. The intracellular mechanism was further identified using genistein, a tyrosine kinase inhibitor (Figure 2(c)). Corresponding to the effect of cycloheximide and monensin, genistein abolished the intermittent mechanical stress-induced transcription of* IGF-1*. Taken together, we concluded that intermittent mechanical stress required intermediate protein related to tyrosine kinase to induce* IGF-1* expression in HPDLs.

### 3.3. TGF-*β*1 Related to Intermittent Mechanical Stress-Induced* IGF-1* Expression

**Figure 3:**
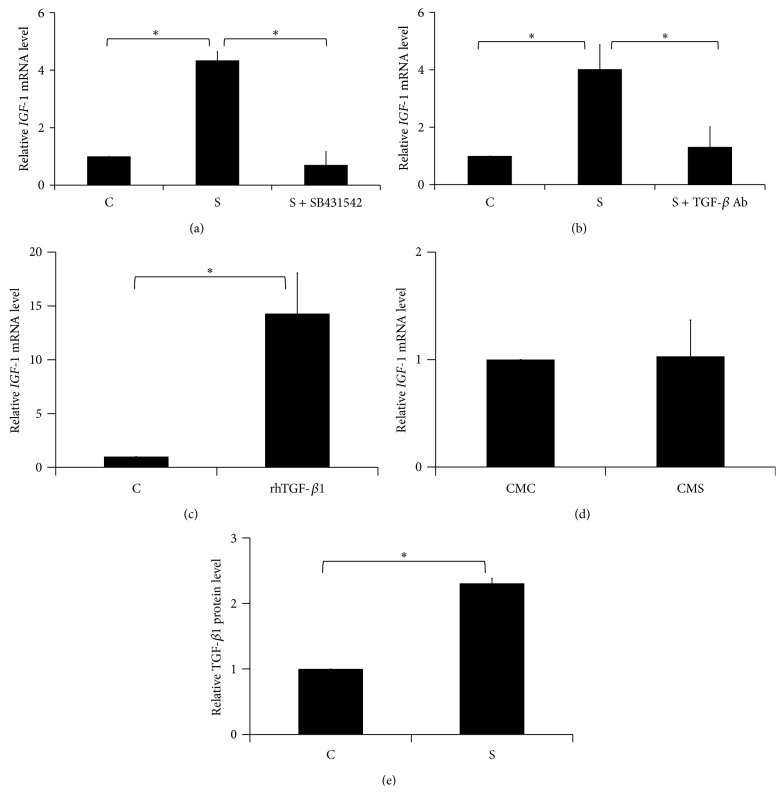
TGF-*β*1 related to intermittent mechanical stress-induced IGF-1 expression. (a) SB431542 (10 *μ*M) or (b) TGF-*β*1 neutralizing antibody (Ab) (5 *μ*g/mL) was used to pretreat HPDLs 30 min before applying the intermittent mechanical stress for 24 h. The* IGF-1* expression was measured by real-time PCR. (c)* IGF-1* mRNA levels were examined after HPDLs were treated with rhTGF-*β*1 (2 ng/mL) for 24 h. (d) HPDLs were treated with cell culture medium from intermittent mechanical stress-treated group (CMS) or untreated group (CMC) 24 h. The* IGF-1* mRNA levels were determined by real-time PCR. (e) The TGF-*β*1 protein in whole cell lysate was measured by ELISA assay. Asterisks indicated statistically significant difference. C: the control condition; S: the intermittent mechanical stress treatment condition.

As described above, the genistein inhibition blocked the intermittent mechanical stress-induced* IGF-1* expression. Thus, SB431542 (TGF-*β* receptor type I (T*β*RI) inhibitor) was chosen to clarify mechanism (Figure 3(a)). The result demonstrated that SB431542 completely suppressed intermittent mechanical stress-induced* IGF-1* mRNA expression. To confirm the TGF-*β*1 role in this phenomenon, the neutralizing antibody against TGF-*β*1 was used to block the binding of TGF-*β*1 and its receptors. Correspondingly with SB431542 treatment, the neutralizing antibody against TGF-*β*1 reduced the IGF-1 transcription under intermittent mechanical stress stimulation (Figure 3(b)). Finally, the addition of exogenous rhTGF-*β*1 resulted in the upregulation of* IGF-1* mRNA levels at 24 h (Figure 3(c)). However, to determine intermittent mechanical stress-induced* IGF-1* expression through TGF-*β*1 protein secretion, we collected the cell culture medium from intermittent mechanical stress-treated group (CMS) as well as the control group (CMC) and transferred it to another set of unstimulated HPDLs for 24 h. Surprisingly,* IGF-1* expression in those cells incubated with CMS-treated group and CMC-treated group did not differ (Figure 3(d)).

Thus, we further measured the protein levels of TGF-*β*1 in both condition mediums and found that TGF-*β*1 protein levels in CMS did not differ from CMC (data not shown). However, the whole cell lysate from intermittent mechanical stress-treated group expressed significantly higher TGF-*β*1 protein levels than the control group (Figure 3(e)). Such evidence may imply intermittent mechanical stress-induced TGF-*β*1 protein to activate* IGF-1* expression in HPDLs.

### 3.4. CoCl_2_ Inhibited the Effect of Intermittent Mechanical Stress on* IGF-1* Expression

**Figure 4:**
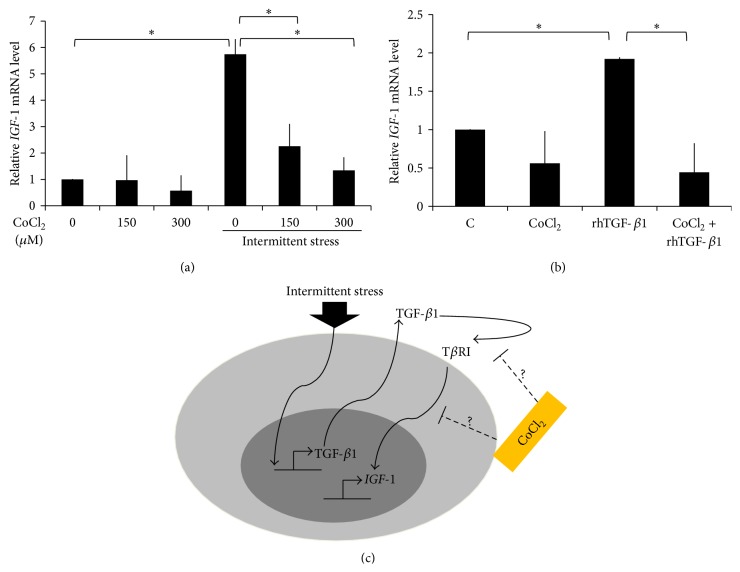
CoCl_2_ inhibited the effect of intermittent mechanical stress on* IGF-1* expression. (a) HPDLs were treated with CoCl_2_ (150 and 300 *μ*M) in the presence or absence of intermittent mechanical stress stimulation for 24 h. The* IGF-1* expression was evaluated by real-time PCR. (b)* IGF-1* expression was examined after HPDLs were treated with rhTGF-*β*1 (2 ng/mL) with and without the CoCl_2_ (150 *μ*M) for 24 h. (c) The illustration represented the proposed signaling mechanism of the intermittent mechanical stress-induced* IGF-1* expression by HPDLs. Asterisks indicated statistically significant difference. C: the control condition; S: the intermittent mechanical stress treatment condition.

Hypoxic condition was mimicked using the CoCl_2_ supplementation at 150–300 *μ*M. The results showed that CoCl_2_ did not significantly affect* IGF-1* expression in normal culture (Figure 4(a)). However, CoCl_2_ significantly inhibited* IGF-1* expression upon the intermittent stress treatment in a CoCl_2_ dose-dependent manner (Figure 4(a)). Further, CoCl_2_ also inhibited the rhTGF-*β*1-induced* IGF-1* expression (Figure 4(b)). Therefore, we hypothesized that CoCl_2_ affect the intracellular signaling of TGF-*β*1 in order to induce* IGF-1* expression in HPDLs.

## 4. Discussion

The physiological force is one of the important factors in maintaining periodontium homeostasis [4]. However, in pathological condition (i.e., periodontal disease), the physiological force may lead to tissue destruction [30, 31]. Thus, this study demonstrated the influence of intermittent mechanical stress on* IGF-1* expression. IGF-1 is an important growth factor regulating cell proliferation and differentiation in HPDLs [23, 24]. We found that the intermittent mechanical stress promoted* IGF-1* expression via TGF-*β*1 signaling pathway. Further, the hypoxic mimic condition using CoCl_2_ could attenuate the intermittent compressive stress-induced* IGF-1* expression, implying that occlusal force may not induce* IGF-1* expression in deep periodontal pocket, where it was considered as hypoxic microenvironment. The suggested model of intermittent mechanical stress-induced* IGF-1* expression in HPDLs is demonstrated in Figure 4(c).

IGF-1 plays an important role in bone growth and development [32, 33] and promotes cell proliferation and osteogenic differentiation in HPDLs [20, 24]. In addition, the* in vitro* study demonstrated that IGF-1 is a growth factor that responds early to mechanical stress [17]. In the* in vivo* orthodontic tooth movement model, the orthodontic force or occlusal stimuli significantly enhanced IGF-1 expression in HPDLs [16]. Correspondingly, our data showed intermittent mechanical stress-induced* IGF-1* expression in HPDLs. However, no evidence explores the detail signaling mechanism of this action. Thus, the present study was the first report which demonstrated that the intermittent mechanical stress promoted* IGF-1* expression by HPDLs through TGF-*β*1 pathway.

The present study showed that the intermittent compressive stress enhanced the increase of TGF-*β*1 protein expression in cell lysate and the addition of rhTGF-*β*1 resulted in the upregulation of* IGF-1* expression similar to those treated with the intermittent mechanical stress. Correspondingly, it was previously demonstrated that the TGF-*β*1 treatment significantly increased IGF-1 expression in dose- and time-dependent manner in human marrow stromal osteoblast precursor cells [34]. It was also shown that single-dose administration of TGF-*β*1 promoted the osteogenic maker expression via the expression of IGF-1 since the knockdown of insulin receptor substrate 1 could attenuate the TGF-*β*1-induced osteogenic marker expression [35]. However, it should be noted that the repeat-dose of TGF-*β*1 led to the inhibition of IGF-1 expression and subsequently caused the suppression of osteogenic differentiation in HPDLs, human mesenchymal stem cells, and murine preosteoblast (MC3T3-E1 cells) [35]. Moreover, TGF-*β* inhibited migration in C2Cl2 skeletal muscle satellite cell and P19 embryonal carcinoma cell via decreasing IGF-1 [36]. Collectively, several lines of evidence indicated the close relationship between TGF-*β*1 and IGF-1 in a positive or negative regulator depending on cell types.

The influence of hypoxia can be found in inflamed tissue including periodontitis. The hypoxic condition is associated with imbalance between elevating the oxygen demand from inflammatory cells penetration and inadequate oxygen supply by poor perfusion [26, 37, 38]. In periodontitis, the HPDLs respond to hypoxia by increasing the inflammatory mediator [39, 40] and enhancement of alveolar bone loss [41, 42]. Therefore, both intermittent mechanical stress and hypoxia are contributing factors to periodontal disease progression, leading us to investigate the effect of combining those two factors on HPDLs. In this study, artificial hypoxic agent, CoCl_2_, abolished the intermittent mechanical stress-induced* IGF-1* expression in HPDLs. This condition represents the clinical situation, where the physiological force was loaded on periodontitis' teeth. Therefore, this data assumed that hypoxia attenuated the intermittent mechanical stress-induced osteogenic differentiation through decrease in* IGF-1* expression in HPDLs. However, the further investigation is indeed required to claim this hypothesis. Recently, it was demonstrated that the cyclic tensile stress under hypoxic condition regulated proliferation and osteogenic differentiation in HPDLs via MAPK pathway [9]. Thus, this information could imply that the type, amount, and direction of force may play an important role in the HPDLs' response under hypoxic condition.

CoCl_2_, an inducer of hypoxia, is well known and commonly employed to establish the physical hypoxic-like condition in cell culture [43–46]. Some studies demonstrated the similar effect of CoCl_2_ and physical hypoxia [47–49]. Our previous study reported that CoCl_2_ could stabilize hypoxia-inducible factor-1 alpha (HIF-1*α*), a key transcription factor for hypoxic condition in HPDLs [50]. In contrast to some lines of evidence, there were some aspects of detailed different mechanisms between hypoxia and CoCl_2_ [51, 52]. Thus, the results from the present study may not be directly implied to those of physical hypoxia setting. Further experiment is required to fully investigate the role of hypoxia on the intermittent compressive stress-induced* IGF-1* expression in HPDLs.

The present study showed that hypoxic mimic condition attenuated the intermittent mechanical stress-induced* IGF-1* expression in HPDLs. However, in unloading condition, CoCl_2_ did not significantly affect* IGF-1* expression. Corresponding to previous studies, physical hypoxia and CoCl_2_ attenuated* IGF-1* expression via the suppression of Runx2 and the induction of C/EBP*δ* in rat osteoblasts [53]. Runx2 could bind to the upstream element in IGF-1 gene promoter and regulated* IGF-1* expression [53]. Further, in systemic investigation, the serum IGF levels were decreased in acute respiratory distress patients, which were a hypoxia state [54]. On the contrary, it was shown that CoCl_2_ decreased* IGF-1* expression in fish muscle [55]. In addition, the IGF-1 expression was upregulated by hypoxia in HepG2 cells [56]. Further, in the present study, we demonstrated that CoCl_2_ inhibited rhTGF-*β*1-induced* IGF-1* expression in HPDLs. The previous study demonstrated that hypoxia inhibited TGF-*β*1-induced transformation in rabbit corneal keratocyte [57]. Taken together, further study to evaluate the mechanism of hypoxic mimic condition on the inhibition of TGF-*β*1-induced* IGF-1* expression in HPDLs is necessitated.

In conclusion, our results indicated the intermittent mechanical stress-induced* IGF-1* expression via TGF-*β*1 signaling pathway in HPDLs. Further, the hypoxic mimic agent could abolish this effect. Our data showed the important intermittent mechanical stress to regulate HPDLs activity.

## Supplementary Material

HPDLs were seeded in 6-well plates at a density of 3×10^5^ cells per well for applying the force and 24-well plates at density of 5×10^4^ cells per well for being treated with CoCl_2_. Subsequently cells were starved with serum-free media 4 h before treatment. At 24 h, HPDLs were incubated with 3-(4, 5-dimethylthiazol-2-yl)-2, 5-diphenyltetrazolium bromide solution for 30 min. Formazan crystals were solubilized in DMSO/glycine buffer solution (0.1 M glycine/0.1 M sodium chloride pH10). The solution was further measured for an absorbance at 570 nm in a microplate reader (Elx800, Biotek, USA). The data were normalized to the control. All measurements were done in triplicate.
