# Supplementary material for: Intermittent Compressive Stress Enhanced Insulin-Like Growth Factor-1 Expression in Human Periodontal Ligament Cells
Source: Int J Cell Biol. 2015 May 28;2015:369874. doi: 10.1155/2015/369874 (PMC4464684; doi:10.1155/2015/369874)
Supplement: Supplementary file 1 — HPDLs were seeded in 6-well plates at a density of 3×10^5^ cells per well for applying the force and 24-well plates at density of 5×10^4^ cells per well for being treated with CoCl2. Subsequently cells were starved with serum-free media 4 h before treatment. At 24 h, HPDLs were incubated with 3-(4, 5-dimethylthiazol-2-yl)-2, 5-diphenyltetrazolium bromide solution for 30 min. Formazan crystals were solubilized in DMSO/glycine buffer solution (0.1 M glycine/0.1 M sodium chloride pH10). The solution was further measured for an absorbance at 570 nm in a microplate reader (Elx800, Biotek, USA). The data were normalized to the control. All measurements were done in triplicate. [file 369874.f1.pdf]

Supplementary Figure Legends

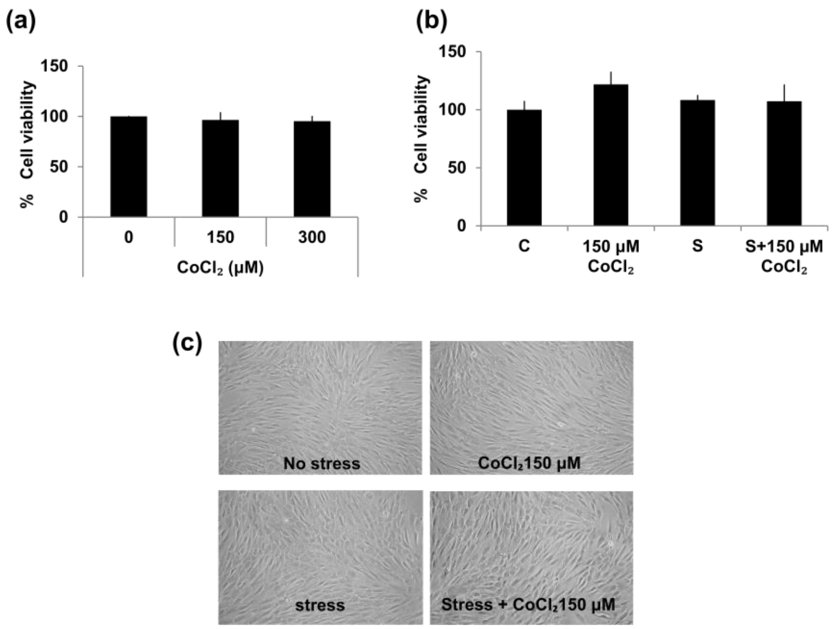

Supplementary figure 1. HPDLs viability upon exposure to $CoCl_2$ or combined with intermittent mechanical stress was evaluated using MTT assay (a&b) and microscopic examination (c; 100X, original magnification) at 24 h

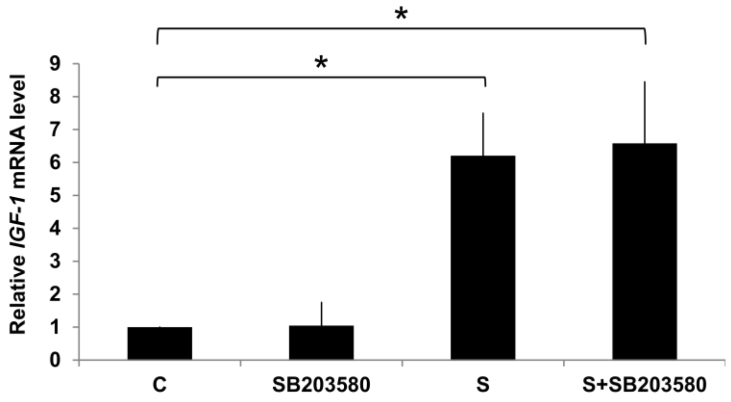

Supplementary figure 2. Intermittent mechanical stress induced *IGF-1* expression independent of p38 signaling pathway. SB203580 (3.5 μM) was used to pre-treat HPDLs 30 min before applying the intermittent mechanical stress for 24 h. The IGF-1 expression was measured by real-time PCR. Asterisks indicated statistically significant. (C; the control condition, S; the intermittent mechanical stress treatment condition)
